# Transcriptomic Analysis Reveals the Growth Regulatory Mechanisms in Diploid, Triploid, and Tetraploid Pacific Oyster (*Crassostrea gigas*)

**DOI:** 10.3390/ani15182691

**Published:** 2025-09-14

**Authors:** Yuting Meng, Yousen Zhang, Weijun Wang, Yancheng Zhao, Daowen Qiu, Zan Li, Guohua Sun, Cuiju Cui, Qiang Wang, Zhongyi Liu, Jianmin Yang

**Affiliations:** 1 School of Fisheries, Ludong University, Yantai 264025, China; 2 College of Fisheries and Life Science, Shanghai Ocean University, Shanghai 201306, China; 3 Yantai Haiyu Marine Technology Co., Ltd., Yantai 264000, China; 4 1.8 Meters Marine Technology (Zhejiang) Co., Ltd., Hangzhou 311100, China

**Keywords:** *Crassostrea gigas*, diploid, triploid, tetraploid, innate immune, growth

## Abstract

Ploidy level exerts profound influences on the phenotypic and physiological traits of *Crassostrea gigas*. Compared to diploids, triploids exhibit desirable characteristics such as sterility, a faster growth rate, and improved meat quality. In contrast, tetraploids often suffer from slow growth, yet the mechanisms underlying these polyploid-associated traits remain unclear. This study aimed to elucidate these mechanisms by comparing differences in growth-related phenotypes and gene expression among diploid, triploid, and tetraploid oysters. We identified 1533 differentially expressed genes (DEGs) between diploids and triploids, 946 DEGs between triploids and tetraploids, and 1326 DEGs between diploids and tetraploids. Through trend analysis, we clustered genes with similar expression changes across ploidy levels and conducted functional enrichment analysis on these gene clusters. The results revealed that genes associated with the innate immune response were significantly up-regulated in tetraploids, whereas genes related to biomineralization and metabolism were markedly up-regulated in triploids. These findings suggest that tetraploid oysters may mount a stronger innate immune response compared to diploids and triploids, while triploids demonstrate superior growth performance. This study provides valuable resources for investigating the functional aspects of genes related to polyploid phenotype differences.

## Simple Summary

Compared to diploids, triploid Pacific oysters (*Crassostrea gigas*) exhibit faster growth and enhanced resilience to environmental stress. While tetraploids are vital for breeding programs, their growth performance is often suboptimal. The molecular mechanisms through which ploidy influences these traits are still not fully understood. In this study, triploid sterility—resulting from abnormal sex hormone levels—redirects energy from reproduction to somatic growth, significantly enhancing shell formation and stress resistance, the latter linked to increased taurine synthesis. In contrast, tetraploids show impaired growth due to the excessive energy consumption of an overactive immune system. These findings deepen our understanding of how ploidy impacts oyster physiology and provide valuable insights for breeding superior oyster strains.

## 1. Introduction

The Pacific oyster (*Crassostrea gigas*) is an ecologically and economically important species in global aquaculture, serving as an ecosystem engineer that provides significant ecological benefits [1,2]. Nevertheless, a major challenge in conventional diploid oyster farming arises during the high-demand summer months, which align precisely with their peak reproductive maturation. During this phase, diploid oysters divert a substantial portion of their energy reserves into gonad development, leading to a considerable decline in sensory quality (taste and flavor), thereby failing to meet the market’s demand for premium oysters [3,4]. The emergence of triploid oysters has transformed this situation. The primary advantage of triploid oysters lies in their impaired gonad fertility or delayed gonadal development. Due to the inability to carry out normal gametogenesis, energy typically devoted to reproduction is redirected toward somatic growth and maintenance. As a result, oysters can be harvested year-round with consistent quality, thereby enhancing the economic efficiency of aquaculture and stabilizing market supply [5,6,7]. The establishment of tetraploid oyster strains has addressed the bottleneck for the large-scale production of triploids, as crossing tetraploids with diploids generates 100% triploid offspring [8,9]. This attribute circumvents the need for laborious artificial triploid induction in each generation, thereby furnishing a solid and dependable technological basis for the mass, consistent, and efficient industrial-scale propagation of triploid oysters [10,11].

The production performance of triploid oysters is generally superior to that of diploids [12,13,14]. This advantage is likely attributable to their sterility, as the absence of reproductive investment allows more energy to be allocated to somatic growth and shell development [3,12]. During aquaculture, the growth rate of tetraploid oysters is slower than that of diploids [15,16], which contrasts with previous findings suggesting that growth performance is generally enhanced by genome duplication [17,18]. The slow growth rate of tetraploids may also be associated with the increased energy required to separate the four sets of chromosomes and develop giant cells during meiosis and mitosis [19]. Extensive research has been conducted on the effects of ploidy level on disease resistance in oysters, which is considered an indirect mechanism by which triploids avoid disease. In fact, the faster growth rate of triploids shortens the duration of potential disease exposure [20]. Moreover, numerous studies have demonstrated that oysters with different ploidy levels exhibit distinct physiological characteristics across seasons, largely due to differences in their reproductive strategies [21]. The intensive gonadal development and spawning activities in fertile oysters cause physiological and metabolic disturbances, making them more susceptible to summer mortality [22,23]. Polyploids differ from diploids in their genomic architecture, which substantially influences the expression of duplicated genes, including gene silencing as well as the up- or down-regulation of duplicate-associated genes. These alterations may involve specific genetic variations or regulatory mechanisms and are likely linked to the trait and phenotypic differences observed in polyploids [24,25].

Extensive research has been carried out to enhance the industrial-scale cultivation of oysters, with particular emphasis on histology, biochemical composition, and genomic variation. Triploid oysters have received considerable attention, especially regarding their physiological differences during gametogenesis [26,27]. In contrast, relatively few studies have addressed the physiological mechanisms underlying variation in other tissues, or examined the influence of ploidy on oyster growth, particularly in tetraploids. This study aims to decipher the molecular mechanisms driving the distinct physiological strategies and growth patterns of diploid, triploid, and tetraploid oysters. It provides a valuable target gene resource for future research on polyploidy and gene function, contributing to a deeper understanding of the molecular mechanisms underlying growth, development, and adaptation in polyploid oysters.

## 2. Materials and Methods

### 2.1. Animal Materials and Sample Collection

All samples of diploid, triploid, and tetraploid *C. gigas* used in this experimental study were provided by the Kongtong Island Breeding Base (Yantai, Shandong Province, China). The study utilized the new variety “LUYI No. 1” (Certification Number: GS-01-006-2020) diploid oysters as the foundation material. Induced triploid oysters were generated by inhibiting the first polar body extrusion during fertilization using “LUYI No. 1”, and tetraploid F1 generation was subsequently induced through mating between female triploid and male diploid oysters. The experimental tetraploid oysters were obtained from the F2 generation generated by mating male and female F1 tetraploids. The triploid oysters used in this study were obtained through interploid hybridization using diploid oysters as females and F1 tetraploid oysters as males. The diploid oysters used in the experiment were produced by mating between male and female diploid oysters. For each reproduction, at least fifty males and females were used. Among the experimental oysters, diploids and triploids share the same maternal lineage, while triploids and tetraploids share the same paternal lineage, demonstrating a well-documented genetic background. The oysters were reared in cages for one year in a natural shallow seawater environment (approximately 6–8 m below sea surface; annual temperature range: 3–26 °C) near Kongtong Island. Each cage layer contained approximately 10 individuals, and the oysters fed on natural marine plankton during the culture period. Sampling was performed in July of the following year.

### 2.2. Ploidy Verification and Measurement of Phenotypic Traits

Before tissue collection, the ploidy level of every oyster was confirmed by flow cytometry (Beckman Coulter Life Sciences, Shanghai, China). Gill filaments were excised and rinsed thoroughly with PBS. Tissues were transferred to eppendorf tubes containing 300 μL PBS and mechanically dissociated with sterile scissors to generate a single-cell suspension. Subsequently, 10 μL of 0.05 mg/mL DAPI (4′,6-diamidino-2-phenylindole dihydrochloride) dye was added for nuclear staining. Samples were briefly vortexed and incubated for 20 min in the dark. The suspension was then filtered through a 300 mesh filter screen and analyzed by flow cytometry for oyster ploidy determination.

For each ploidy group, fifty 1-year-old oysters were randomly selected for phenotypic trait measurements. Phenotypic measurements—including shell length, shell width, and shell height—were recorded for each individual using digital calipers with a precision of 0.01 mm. Total weight was measured for every specimen employing an electronic balance accurate to 0.01 g. Following this, adductor muscles were dissected from nine selected oysters per ploidy level. Tissues were rinsed with phosphate-buffered saline (PBS) and sectioned into fragments. Samples were promptly snap-frozen in liquid nitrogen, and stored at −80 °C for subsequent RNA isolation for transcriptomic sequencing.

### 2.3. Total RNA Extraction, cDNA Library Construction and Sequencing

Total RNA isolation from *C. gigas* adductor muscles was performed with Trizol reagent (Invitrogen, Carlsbad, CA, USA) according to the manufacturer’s instructions. RNA integrity was rigorously assessed using an Agilent 2100 bioanalyzer (Agilent Technologies, Santa Clara, CA, USA). Following quality confirmation, a portion of each RNA sample was used for library preparation, while the remainder was reserved for quantitative RT-PCR (qRT-PCR) verification. Library construction methodology followed established procedures from prior studies. Briefly, equimolar amounts of RNA from every three biological samples within each experimental group were pooled. This pooling strategy was repeated to generate three independent pooled samples per group [28,29,30]. These pooled samples were used for library construction. Following library qualification, sequencing was conducted using the illumina NovaSeq 6000 (Illumina, San Diego, CA, USA).

### 2.4. Data Processing and Analysis

The raw data were firstly filtered. In this step, clean data were obtained by removing reads containing adapter, reads containing ploy-N (N denotes an undetermined base in nucleotide sequencing) and low quality reads from raw data. At the same time, Q20, Q30 and GC content of the clean data were calculated. All the downstream analyses were based on the clean data with high quality. A reference genome index was built with HISAT2 v2.0.5, followed by alignment of paired-end clean reads to the reference genome employing HISAT2 v2.0.5.

### 2.5. Analysis of DEGs

Differential expression analysis between experimental groups was performed using DESeq2. Differentially expressed genes (DEGs) were defined as those meeting a *p*-value ≤ 0.05 and |log2 (fold change)| ≥ 1 [31]. Functionally significant DEGs were mapped to Gene Ontology (GO) terms and Kyoto Encyclopedia of Genes and Genomes (KEGG) pathways using DAVID v6.8. DEGs demonstrating significant expression trends were then subjected to clustering analysis.

### 2.6. qRT-PCR Verification

qRT-PCR was utilized to verify the reliability of RNA-seq data. Primers were designed using Prime Premier 5.0 software based on transcript sequences, with EF-1α serving as the reference gene for expression level normalization. The primer sequences are provided in Table 1. Twelve DEGs were selected for validation.

## 3. Results

### 3.1. Ploidy Analysis and Phenotypic Traits

**Figure 1:**
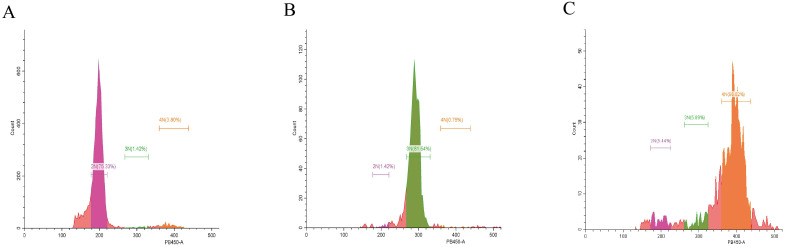
Flow cytometric analysis of ploidy levels of *C. gigas* in this experiment. (**A**–**C**) reveal the characteristic DNA content peaks representative of diploid, triploid, and tetraploid oysters, respectively.

**Figure 2:**
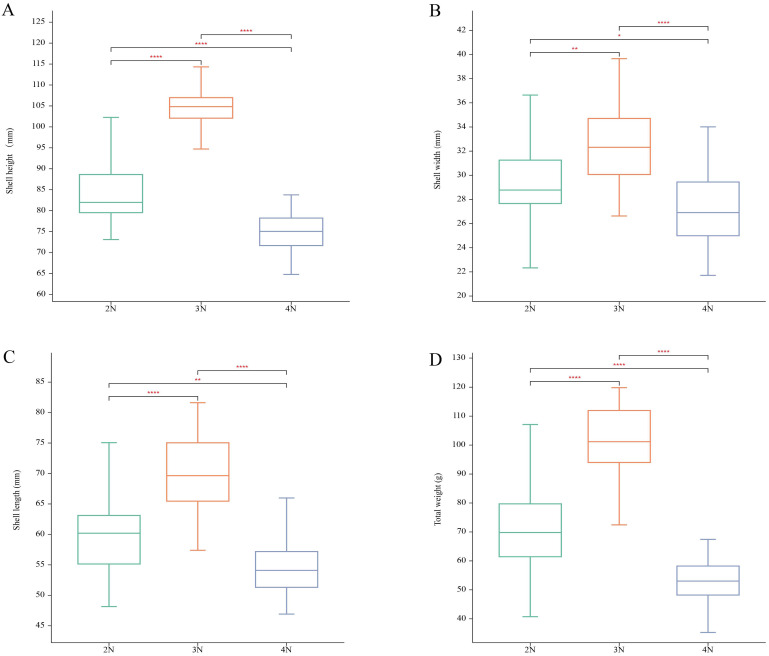
Analysis of phenotypes in *C. gigas* between 2N (diploid), 3N (triploid), and 4N (tetraploid) oysters. (**A**) Shell height; (**B**) Shell width; (**C**) Shell length; (**D**) Total weight. The symbol “*” indicates a difference among the three groups at the *p* < 0.05 level; “**” indicates a significant difference among the three groups at the *p* < 0.01 level; “****” indicates a significant difference at the *p* < 0.0001 level.

The ploidy levels of all oysters were confirmed by measuring blood cell DNA content via flow cytometry. Since nuclear DNA is stained by DAPI fluorescent dye, and fluorescence intensity is proportional to cellular DNA content, the relative DNA content of the tested cell populations could be determined using the known diploid DNA content as a reference. The DNA content of triploid and tetraploid cells is 1.5 times and 2 times that of diploid cells, respectively (Figure 1). Phenotypic traits including shell length, shell width, shell height, and total body weight were analyzed across oysters of different ploidy levels (Figure 2). Triploids exhibited significantly greater values for all measured traits compared to both diploids and tetraploids. Tetraploids displayed significantly reduced shell dimensions and body weight relative to diploids. These results indicate a pronounced growth advantage in triploids, while tetraploids show the opposite pattern.

### 3.2. Assembly of Sequencing Data

The average numbers of clean reads for diploid, triploid, and tetraploid samples were 43,355,229, 45,147,518, and 44,476,049, respectively. The single-end reads from each library were aligned to the reference genome of *C. gigas*, and 81.92%, 82.69%, and 81.20% of the reads from diploid, triploid, and tetraploid libraries, respectively, were successfully mapped to the reference genome. Among these reads, 77.30%, 78.34%, and 76.57% were uniquely mapped reads. The GC contents for diploid, triploid, and tetraploid samples were 44.51%, 44.53%, and 44.47%, respectively. Subsequently, the total number of clean reads for all ploidy levels showed a Q20 percentage greater than 97.41% and a Q30 percentage greater than 92.88% (Table 2).

### 3.3. DEGs Expression

**Figure 3:**
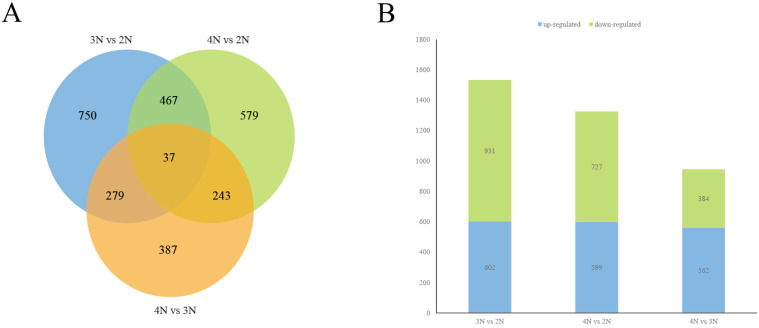
Venn diagram and histogram of the number of DEGs. (**A**) Venn diagram of the number of DEGs. (**B**) Histogram showing the number of DEGs that were up- or down-regulated between different groups.

Figure 3 illustrates that 1533 genes exhibited significant differential expression in 3N vs. 2N, with 504 DEGs shared with the 4N vs. 2N group and 316 DEGs overlapping with the 4N vs. 3N contrast. Notably, 750 DEGs were uniquely identified in the 3N vs. 2N comparison, whereas 37 DEGs were conserved across all three comparison groups. In the 4N vs. 2N analysis, 1326 significant DEGs were identified, of which 280 were shared in the 4N vs. 3N comparison, and 579 DEGs were exclusive to the 4N vs. 2N comparison. For 4N vs. 3N, 946 significant DEGs were observed, including 387 DEGs specific to this pairwise comparison. Within the 3N vs. 2N comparison, 602 genes showed up-regulation and 931 demonstrated down-regulation, while 599 up-regulated and 727 down-regulated genes were detected in 4N vs. 2N. Furthermore, the 4N vs. 3N contrast displayed 562 up-regulated and 384 down-regulated genes.

### 3.4. Functional Annotation and Enrichment Analysis of DEGs

**Figure 4:**
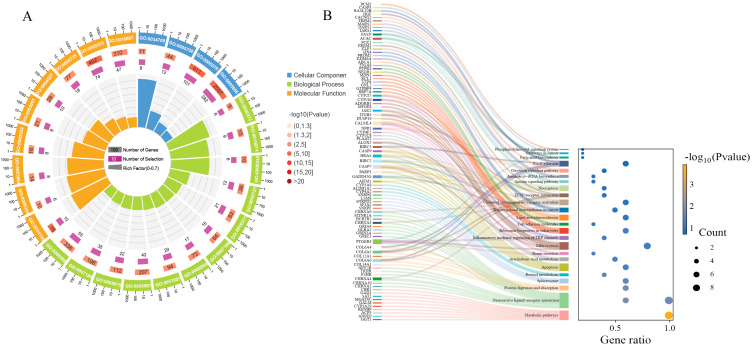
The GO (**A**) and KEGG (**B**) enrichment of DEGs. (**A**) GO:0034709: methylosome, GO:0005758: mitochondrial intermembrane space, GO:0005576: extracellular region, GO:0005886: plasma membrane, GO:0000272: polysaccharide catabolic process, GO:0015074: DNA integration, GO:0006032: chitin catabolic process, GO:0050727: regulation of inflammatory response, GO:0016311: dephosphorylation, GO:0007189: adenylate cyclase-activating G protein-coupled receptor signaling pathway, GO:0006955: immune response, GO:0051607: defense response to virus, GO:0045087: innate immune response, GO:0005975: carbohydrate metabolic process, GO:0007186: G protein-coupled receptor signaling pathway, GO:0006508: proteolysis, GO:0004190: aspartic-type endopeptidase activity, GO:0008391: arachidonic acid monooxygenase activity, GO:0003964: RNA-directed DNA polymerase activity, GO:0042562: hormone binding, GO:0005001: transmembrane receptor protein tyrosine phosphatase activity, GO:0004527: exonuclease activity, GO:0004725: protein tyrosine phosphatase activity, GO:0005509: calcium ion binding, GO:0016887: ATP hydrolysis activity.

Functional annotation of all DEGs in the three comparison groups was performed through GO (Figure 4A) and KEGG (Figure 4B) enrichment analyses. In the GO analysis, DEGs were assigned to the three core GO categories: biological process (BP), cellular component (CC), and molecular function (MF). Within biological process category, terms such as innate immune response, DNA integration, and polysaccharide catabolic process were among the most significantly enriched. Cellular component analysis highlighted plasma membrane, methylosome, and mitochondrial intermembrane space as key enriched terms. Molecular function category revealed calcium ion binding, protein tyrosine phosphatase activity, and ATP hydrolysis activity as highly enriched terms. KEGG pathway analysis indicated significant enrichment for DEGs in multiple pathways such as metabolic pathways, neuroactive ligand–receptor interaction, and insulin signaling pathway, providing mechanistic insights into ploidy-dependent variations in growth and development, immune responses, and metabolic processes across diploid, triploid, and tetraploid oysters.

### 3.5. Expression Trend Profiling

**Figure 5:**
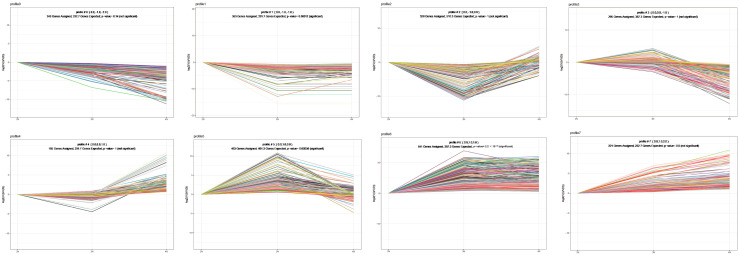
Series Test of Cluster. The lines in different colors represent distinct genes. The number of genes and *p*-values for different ploidy gene expression patterns are shown in the figure.

**Figure 6:**
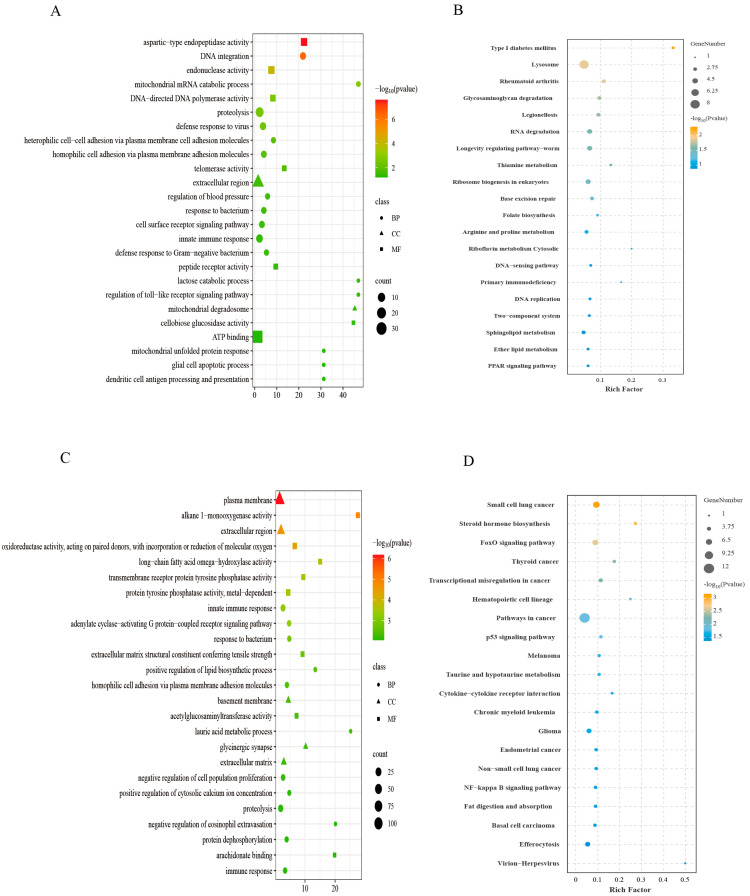
GO and KEGG Bubble plots. GO (**A**) and KEGG (**B**) bubble plots of DEGs in profile 2 and profile 4. GO (**C**) and KEGG (**D**) bubble plots of DEGs in profile 3 and profile 5.

For enhanced classification of DEGs by ploidy level, trend analysis was conducted on all DEGs to delineate expression patterns correlating with ploidy-dependent phenotypic and physiological alterations. DEGs were clustered into eight distinct expression profiles (Figure 5). Among them, profile 2, 3, 4, and 5 exhibited expression profiles associated with ploidy-dependent physiological characteristics. In profiles 2 and 4, tetraploid oysters demonstrated peak expression intensities, whereas triploid expression was either lower than or equivalent to diploid levels. Conversely, in profiles 3 and 5, triploids exhibited the highest expression levels, with tetraploid expression being lower than or equal to diploid levels. To functionally characterize these DEGs, GO and KEGG enrichment analyses were performed on DEGs in these profiles. DEGs in profiles 2 and 4 showed significant enrichment for immune-associated pathways, such as defense response to virus, response to bacterium, innate immune response, lysosome, and primary immunodeficiency (Figure 6A,B). DEGs in profiles 3 and 5 were primarily enriched in growth and metabolism-related pathways, including proteolysis, steroid hormone biosynthesis, taurine and hypotaurine metabolism, and cytokine–cytokine receptor interaction (Figure 6C,D).

### 3.6. Validation of RNA-Seq by qRT-PCR

**Figure 7:**
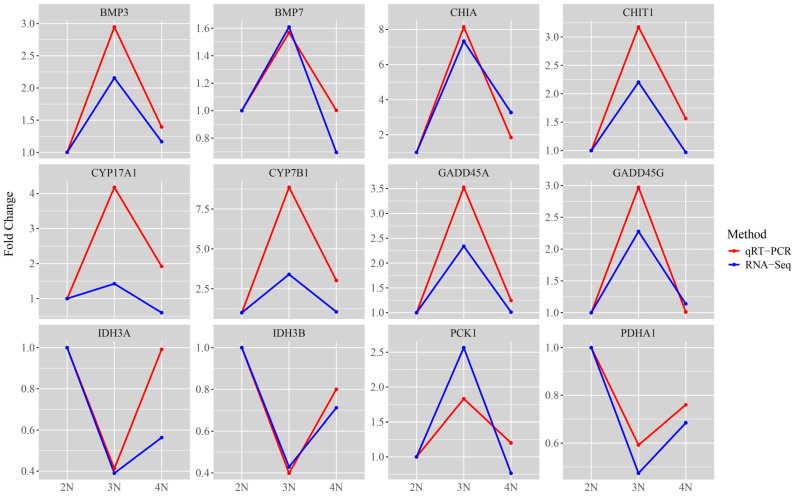
qRT-PCR and RNA-Seq results of DEGs.

Gene expression accuracy was verified through qRT-PCR. The gene expression patterns obtained by qRT-PCR were consistent with those derived from RNA-Seq analysis, confirming the reliability of RNA-Seq findings (Figure 7).

## 4. Discussion

### 4.1. Trend Analysis Among Different Ploidy Levels

To further investigate the patterns of phenotypic and physiological traits associated with ploidy variation, we performed trend analysis on differentially expressed genes (DEGs) and conducted GO and KEGG enrichment analyses on DEGs with correlated expression patterns. In the functional enrichment results of DEGs from profiles 2 and 4, terms related to innate immune response—such as defense response to virus, antibacterial response, innate immunity, lysosomal activity, and primary immunodeficiency—were significantly enriched. These pathways are closely associated with innate immune function. Genes associated with the innate immune response exhibited the highest expression in tetraploids and the lowest in triploids, suggesting potential overexpression of these genes in tetraploids, which is consistent with our previous findings [32]. The activation and maintenance of innate immune responses constitute an energy-intensive process [33]. This necessitates substantial energy investment in signaling molecule biosynthesis, effector protein production, and heightened immune cell activities (activation, proliferation, migration, and phagocytosis) [34]. Sustaining the innate immune system in a state of “high alert” or “overactivation” necessitates continuous and significant allocation of limited energy resources. While this redistribution is crucial for immune development, function, and regulation, it inevitably incurs trade-offs. Biological growth—including cell proliferation, tissue building, protein synthesis, and glycogen storage—is itself an energy-intensive process. When resources are disproportionately and persistently directed toward immune defense, the energy available for supporting somatic growth and maintaining energy reserves becomes relatively scarce, thereby exerting a negative impact on organismal growth. Moreover, elevated expression of these genes could lead to cytotoxic effects or dysregulated immune responses, impairing key cellular and systemic functions, and potentially inducing immunosuppression or autoimmune disorders [7,35]. These may offer novel insights into the slower growth rate observed in tetraploids.

In profiles 3 and 5, DEGs were significantly enriched in terms such as steroid hormone biosynthesis, taurine and hypotaurine metabolism, and cytokine–cytokine receptor interaction, with expression levels notably higher in triploids than in diploids and tetraploids. This phenomenon may provide key molecular insights into the reproductive sterility, rapid growth rate, and superior environmental adaptability of triploid oysters. Furthermore, following whole-genome duplication, some genes related to growth, reproduction, and environmental adaptation exhibit negative dosage effects [36,37]. This suggests that tetraploid organisms employ complex regulatory mechanisms—such as genomic methylation, chromatin restructuring, and other epigenetic modifications—in response to additional gene copies [38,39]. Consequently, certain genes may be subject to negative dosage effects regulation, resulting in their reduced expression in tetraploids and thereby influencing growth, reproduction, and adaptive capacity.

### 4.2. Innate Immune Response in Tetraploid C. gigas

As filter feeders inhabiting microbially dense marine environments, oysters are constantly exposed to millions of potential pathogens. Innate immunity serves as the first line of host defense against foreign pathogen invasion, which activates downstream interferon signaling through nonspecific receptors recognizing conserved pathogenic patterns (located on cell surfaces, within endosomes, and in the cytoplasm). This initiates robust immune responses to combat infection and alert neighboring cells. However, over-activation of the immune response can lead to significant consumption of energy resources and may trigger persistent inflammation and tissue damage, thereby increasing morbidity and mortality [40]. Consequently, precise regulation of innate immunity is critical for balancing inflammatory signals and maintaining cellular homeostasis.

**Figure 8:**
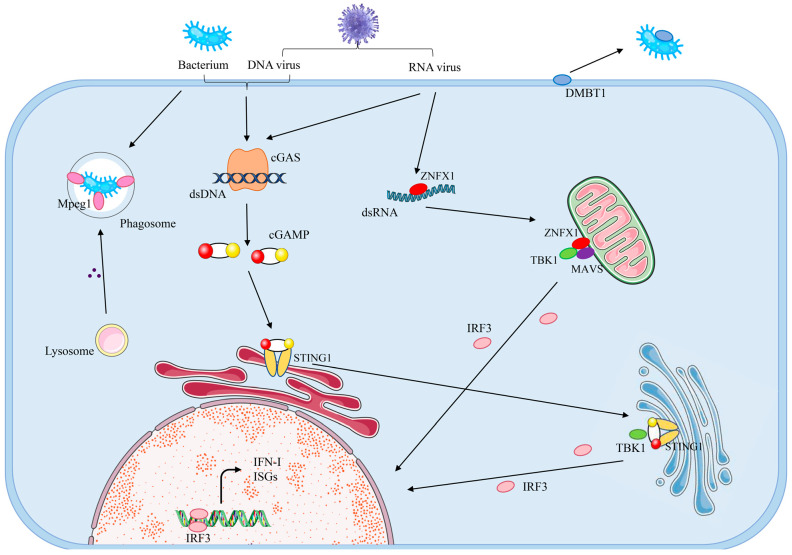
Molecular mechanisms associated with innate immunity in tetraploid oysters.

In our study, we investigated the molecular basis of immune hyperactivation in tetraploid oysters (Figure 8). Cyclic GMP-AMP synthase (cGAS), an innate immune sentinel for aberrant cytosolic double-stranded DNA (dsDNA), detects abnormal dsDNA and plays a crucial role in innate immune responses. Following recognition and binding of abnormal dsDNA, activated cGAS synthesizes the secondary messenger cGAMP from ATP and GTP. cGAMP then binds to stimulator of interferon interactor 1 (STING1) on the endoplasmic reticulum (ER), inducing conformational changes that facilitate STING1 translocation from ER to Golgi apparatus. There, STING1 recruits TANK-binding kinase 1 (TBK1). TBK1 phosphorylates STING1 at its C-terminal domain and facilitates recruitment of interferon regulatory factor 3 (IRF3). Phosphorylated IRF3 dimerizes and translocates to the nucleus, initiating transcription of type I interferons (IFNs) and interferon-stimulated genes (ISGs) with antiviral activities, thereby activating downstream immune responses. Additionally, zinc finger NFX1-type containing 1 (ZNFX1) recognizes and binds double-stranded RNA (dsRNA) from RNA viruses, then associates with mitochondrial antiviral signaling protein (MAVS), similarly initiating TBK1-dependent signaling that phosphorylates IRF3, driving the expression of type I IFNs and ISGs for immune activation [41,42]. Type I IFNs (predominantly IFN-α/β) and ISGs, produced by all infected cells, are essential for establishing an antiviral state. They directly inhibit viral replication or regulate immune responses to constrain viral spread, conferring antiviral protection to adjacent cells [43]. However, overproduction of type I IFNs and ISGs may provoke tissue injury and autoimmunity [44,45].

Research demonstrates that Macrophage-expressed Gene 1 (Mpeg1/Perforin-2) functions as a critical antimicrobial pore-forming protein. As a principal effector of the innate immune system, it is essential for eliminating bacteria following phagocytosis [46,47]. During bacterial infection, it translocates and polymerizes on bacterial surfaces to form pores, compromising bacterial envelope integrity and enhancing susceptibility to lysozyme-mediated degradation [48]. Bacteria-induced upregulation of Mpeg1 has been documented in diverse mollusks, including abalone and oysters [49,50]. Furthermore, the glycoprotein DMBT1 is a high-molecular-weight secreted epithelial glycoprotein involved in innate immune defense. It attenuates bacterial virulence by binding to fimbriae, thereby impairing bacterial motility and adhesion. This mechanism consequently blunts bacterial pathogenicity and safeguards host cells against microbial assaults [51,52].

In summary, we propose that the heightened expression of these genes in tetraploid oysters may enable a stronger innate immune response compared to diploid and triploid oysters when challenged by various pathogens. This enhanced immune activity likely diverts energy resources toward immune defense, thereby reducing the energy available for growth and resulting in the smaller body size observed in tetraploids.

### 4.3. Sterility Environmental Adaptability, and Enhanced Growth Mechanisms in Triploid C. gigas

#### 4.3.1. Effects of Steroid Hormone Biosynthesis on Sterility in Triploid *C. gigas*

Steroid hormones act as central regulators of invertebrate reproduction [53]. Notably, the biosynthesis of genes critical for sex steroid hormone biosynthesis was greatest expression in triploid oysters (compared to diploids and tetraploids). CYP17A1, a cytochrome P450 family member, plays an essential role in the steroidogenic pathway that produces androgen and estrogen [54], and has been demonstrated to regulate sex differentiation and gonadal development via androgen and estrogen catalysis in carp and zebrafish [55,56]. As a steroid hydroxylase, CYP7B1 modulates estrogen and androgen signaling, affecting cellular levels of androgen and estrogen [57,58]. Altered androgen and estrogen levels impact sex determination, impair gonadal maturation, and suppress reproductive capacity [59]. Overexpression of these genes in steroidogenic pathways in triploids may cause aberrant androgen and estrogen levels and signaling transduction, perturbing normal sex differentiation, impeding gonad development, and ultimately suppressing reproductive take place. This could be an important underlying mechanism for the sterility observed in triploid oysters. The sterility of triploid oysters may allow them to allocate more energy to growth, thus sustaining growth even during the breeding season.

#### 4.3.2. Effects of Taurine and Hypotaurine Metabolism on Environmental Adaptability in Triploid *C. gigas*

Taurine, the most abundant free amino acid in *C. gigas* [60], is critical for tissue development, growth, and physiological maintenance [61]. It enhances growth performance, antioxidant defenses, and environmental stress tolerance [62,63]. For instance, taurine enhances mitochondrial bioenergetics in *C. gigas* under hyperosmotic stress [62]. High taurine accumulation constitutes an evolutionary adaptation in intertidal oysters, serving as a key adaptive strategy against environmental stressors such as aerial exposure and thermohaline fluctuations [60,64]. Critically, taurine deficiency has been shown to reduce growth rates and feed efficiency, even increasing mortality [65,66]. Cysteine dioxygenase 1 (CDO1) catalyzes taurine biosynthesis via the cysteine pathway, acting as a key rate-limiting enzyme in taurine biosynthesis [67]. Beyond this pathway, hypotaurine can be oxidized to taurine by flavin-containing monooxygenase (FMO), constituting a major biosynthetic route essential for endogenous taurine production [68]. In this study, genes involved in taurine synthesis were expressed at significantly higher levels in triploid oysters compared to diploid and tetraploid oysters, suggesting that triploids may possess enhanced capabilities in environmental adaptation and energy utilization, thereby conferring greater resilience to environmental stress.

#### 4.3.3. Effects of the Bone Morphogenetic Protein (BMP) Gene Family on Growth in Triploid *C. gigas*

As ligands of the transforming growth factor-β (TGF-β) superfamily, BMPs function not only as key osteoinductive factors [69], but also regulate critical biological processes including cell proliferation/differentiation, tissue repair, and organogenesis [70,71,72]. In bivalves, BMP3 and BMP7 directly regulate biomineralization of nacreous and prismatic shell layers, playing a critical role in shell formation and structural maintenance [73,74]. Furthermore, the BMP family genes have been implicated in growth regulation in *C. gigas* [75], but its specific regulatory mechanisms are still not fully elucidated. Significantly, in this study, BMP3 and BMP7 were highly expressed in triploid *C. gigas*. This elevated expression may enhance shell biomineralization, explaining the faster shell growth rate observed in triploids. Additionally, as critical growth-regulating factors, the activation of BMPs is likely to broadly promote somatic cell proliferation and growth in triploid oysters.

### 4.4. Limitations and Future Perspectives

This study provides insights into the molecular mechanisms underlying ploidy-dependent phenotypic variation; however, several limitations should be considered. Firstly, our transcriptomic analysis was confined to the adductor muscle. Although this tissue is a crucial metabolic organ central to growth, analyzing tissues directly responsible for reproduction and energy metabolism—such as the gonads and hepatopancreas—would offer a more comprehensive understanding of the systemic physiological trade-offs associated with polyploidy. To provide a broader context, we have previously published transcriptomic data on the mantle tissue of these polyploid oysters [32], and the gonadal transcriptome data from the same cohorts are currently under analysis and will be reported in the near future.

Secondly, while triploid sterility is a well-documented and prevalent phenomenon that forms the basis of our discussion, emerging studies have reported varying degrees of gametogenesis and even partial fertility in triploid oysters [27,76]. Although the “LUYI No. 1” triploid oysters used in this study displayed complete sterility under our conditioning, the potential influence of genetic background and environmental factors on this trait warrants further investigation. Future research should incorporate detailed histological monitoring across different seasons and environments to better understand the determinants and prevalence of reproductive effort in triploid oysters, which is crucial for predicting their long-term performance in aquaculture.

## 5. Conclusions

In this study, we systematically investigated the molecular mechanisms underlying phenotypic variation among diploid, triploid, and tetraploid oysters through trend analysis of differentially expressed genes and functional enrichment. The main findings of this study are:

1. Tetraploid oysters exhibited marked overexpression of genes related to innate immunity (STING1, ZNFX1, Mpeg1, etc.). This hyperactivation of the innate immune response appears to demand diversion of energy resources away from growth and metabolism toward immune defense. Although this response may enhance pathogen resistance, the associated energetic cost is likely a primary contributor to the observed growth retardation in tetraploids.

2. Triploid oysters achieve enhanced growth performance through a coordinated multi-mechanism strategy: Overexpression of genes involved in steroid hormone biosynthesis (CYP17A1, CYP7B1) disrupts reproductive development, resulting in functional sterility and redirecting energy toward somatic growth; elevated expression of taurine synthesis-related genes (CDO1, FMO5) enhances antioxidant capacity and osmoregulatory function, thereby improving environmental stress tolerance; upregulated expression of biomineralization genes such as BMP3 and BMP7 promotes shell formation, directly accelerating growth rates.

Overall, this study offers novel insights into the trade-offs between immunity and growth in tetraploids, and elucidates key genetic drivers of advantageous traits in triploids. These results establish a valuable resource for future studies on polyploidy and gene function, and contribute to a deeper understanding of the molecular mechanisms governing growth, development, and adaptation in polyploid oysters.

## Figures and Tables

**Table 1:** List of primers used for qRT-PCR verification.

Gene Name	Forward Primer (5′-3′)	Reverse Primer (5′-3′)
EF-1α	AGTCACCAAGGCTGCACAGAAAG	TCCGACGTATTTCTTTGCGATGT
BMP3	GGCACAAAGCGAAAGCGGAAAC	GCCAAGTGTTCGGGACCTCATG
BMP7	CCGAAGCCGAAATCTAGCACCAC	CCACGCTGTCGCCTACTGTAAC
PDHA1	CGAGAGTACGCCCTGAACAATGG	ACGGTGATTGGGTCCCTGGTC
IDH3A	CTGCTGCTCAGTGCGGTCATG	AGACGAGCCTCCTAAGTCACCAG
IDH3B	GTCTTCCAAGCCGCAGGAGTTC	CTGGCTGGGCGTGCTGATAATG
PCK1	TGCTCCTATGGAAGCGGATACGG	CAAGCCAGCCCTCTCTCCTACC
GADD45A	GCTGGAGAACAAGTCGGACGATG	GGTCGGTGTGAGAGGACAGGATC
GADD45G	CAAGGAGCCTGTGTTGGTAAGCC	GCCATGCACCGACGACTCTG
CHIT1	GGCGTCAGAGTCGGGCATTTC	TGTGGGTGGTGGGTAAGGAGAC
CHIA	CAACGGACTGGATGCGAGGAATG	TCCACCCACCCACAGCCAATAG
CYP17A1	GGAAGAAGACGAGCTGCCGAATC	GGGGAAACCGACAGGAAGTATGC
CYP7B1	GTACCCACCAGCAATCCACAAGG	ACGTGGCATCAACGAACCTGTC

**Table 2:** Transcriptome sequencing statistics and average read counts mapping to the *C. gigas* genome for diploids, triploids, and tetraploids.

Sample	2N	3N	4N
Clean reads	43,355,229	45,147,518	44,476,049
Raw reads	44,128,517	45,834,319	45,245,143
Mapped reads	35,513,324	37,335,903	36,124,735
Mapping rate (%)	81.92	82.69	81.20
Uniquely mapped reads	33,510,189	35,367,460	34,060,315
Uniquely mapped rate (%)	77.30	78.34	76.57
G/C content (%)	44.51	44.53	44.47
% ≥ Q20	97.41	97.46	97.44
% ≥ Q30	92.88	92.98	92.94

## Data Availability

Data will be made available on request.
